# The Early Detection of Tuberculosis of the Bladder

**Published:** 1907-04-13

**Authors:** 


### Points in Diagnosis.

### THE EARLY DETECTION OF TUBERCULOSIS OF THE BLADDER.

Tuberculous ulceration of the bladder has often been regarded as (1) rare, (2) almost incurable. Modern clinical methods have shown, however, that neither of these characters is true. When looked for, the lesion is found to be not at all uncommon; and, far from being incurable, there is increasing evidence to show that the lesion often gets well by itself.

### The Classical Symptoms.

The chief reason why the lesion used to be thought both rare and incurable was that the symptoms as described in text-books are not the symptoms merely of tuberculous ulceration of the bladder, but are those of this condition *in its late stages.* They are: Pain in the hypogastric region, often paroxysmal in character, and frequently referred also either to the perineum or to the base or tip of the penis; pyuria more or less constant; hæmaturia, usually held to be comparatively slight in amount, and infrequent; and, above all, inability to hold the water for any length of time, owing to the pain produced by distension of the infected bladder, and consequent frequency of micturition, attracting attention, mostly at night, because of the disturbance to sleep; the frequency of micturition differs from that due to granular kidney, for example, in that only an ounce or two of urine may be passed at each time.

These symptoms are obviously due to extensive ulceration of the bladder, with exposure of the nerves in the submucous coat, and inflammation which has led to contracture of the viscus. We will at present confine our attention to the bladder, and we would repeat what we have said above, that the classical symptoms of tuberculous ulceration of the bladder are *late* symptoms; if we wait for all of them to present themselves we allow the condition to pass beyond the curable stage. No physician would wait until there was profuse nummular sputum, with cavernous breathing at one or more apices of the lungs, before he diagnosed phthisis; to wait for the full development of all the symptoms of vesical tuberculosis is to wait until the disease in the bladder has reached the stage corresponding to cavitation in the lung. It is the duty of all of us to try and make the diagnosis much earlier in the case of the bladder, just as in the case of the lung; early diagnosis may save many patients from a painful and slow death.

### The Earliest Symptoms of Tuberculosis of the Bladder.

The symptom of all others which should never be passed by without every effort being made to determine its exact cause is *frequency of micturition.* This frequency must not be due to more than the usual amount of urine being formed, as is the case, for example, in diabetes mellitus, diabetes insipidus, granular kidney, arteriosclerosis, and functional disorders of the nervous system; but to inability to hold more than a small amount at a time. In an old man this symptom would indicate enlargement of the prostate with residual urine; in a young person inflammation of the bladder is the most likely cause, and if the nature of the inflammation is not obvious it is very likely to be tuberculous.

Another, but less common, early symptom may be *hæmorrhage.* The two remaining symptoms—pain and pyuria—may each of them be the first symptom of the lesion; they are more likely to lead to the discovery of the nature of the lesion than is frequency of micturition with the passage of comparatively small amounts of urine each time, or hæmorrhage.

### How to Discover the Nature of the Lesion.

There are three chief means of determining the tuberculous nature of the bladder trouble, and these are:—(1) Staining the centrifugalised deposit of the urine for tubercle bacilli; (2) determining the tuberculo-opsonic index of the patient, with or without injections of minute doses of tuberculin; (3) cystoscoping the bladder.

It is obvious that the first of these three is by far the simplest and least expensive. It is, moreover, extremely efficacious in most cases. One is apt to conclude that to detect tubercle bacilli in urine is both difficult and uncertain; in experienced hands, however, this is by no means the case. *Tubercle bacilli have not infrequently been detected in apparently clear urines.* The fact that a urine contains no pus is, therefore, no proof that it should not be examined for tubercle bacilli. When tubercle bacilli cannot be found, then opsonic index estimations may be of assistance; and in any case, the cystoscope is invaluable in determining the extent of the lesion. A boy of 17 recently came up, complaining of "not feeling quite well, and of having to get up twice each night to pass a little urine." So indefinite were his symptoms that some thought he had no lesion at all. The urine was examined for tubercle bacilli, but none could be found. The tuberculo-opsonic index of the boy's blood was estimated, and turned out to be 1.5. This made a tuberculous lesion somewhere almost certain. The cystoscope was used, and large numbers of nearly-healed tuberculous ulcers were seen coated with phosphatic deposit. The condition was healing by itself, and the case taught at least three things:—

(1) That the symptoms of tuberculous bladder may be very slight indeed; (2) that the lesion may in such cases be very easily missed altogether, so that it is probably more common than recognised; (3) the condition, far from being incurable, may get well by itself, and is therefore amenable to treatment if every effort is made to detect it sufficiently early.

